# Research progress of measuring tools for nursing students’ clinical learning environment

**DOI:** 10.3389/fmed.2025.1458823

**Published:** 2025-03-24

**Authors:** Yun Xu, Qing Wang, Qi Wei

**Affiliations:** School of Nursing, Nanjing University of Chinese Medicine, Nanjing, China

**Keywords:** nursing students, clinical learning environment, measuring tools, research progress, reviewed

## Abstract

**Objective:**

To understand the current situation and progress of clinical learning environment measurement tools for nursing students, this paper reviews the relevant research on clinical learning environment measurement tools for nursing students.

**Methods:**

Three databases (Web of Science, PubMed, and CNKI) were searched for relevant articles. Research articles that met specific criteria were included, with identified articles initially screened by title and keyword. Then the abstracts were screened for relevance, and the full text was read for validation before inclusion. Descriptive analysis was performed with relevant findings from data retrieved from various sources.

**Results:**

We included 19 articles that met the criteria, and introduced nine measurement scales, which completed the reliability and validity test through empirical research, providing an important reference for the assessment of clinical learning environment for nursing students. The Clinical Learning Environment and Supervision instrument (CLESI) has been translated into many languages and is widely used.

**Conclusion:**

This review includes multiple clinical learning environment measurement tools for nursing students, which have important value in evaluating the clinical environment of nursing students and can provide reference for scholars to carry out relevant research and practice. It also introduces the research prospects in this field, aiming to inspire future research.

## Introduction

Nursing is essentially a practical discipline, and clinical learning is a core link in the training of nursing students. The clinical learning environment has an essential impact on the training of nursing students.

The learning environment is a crucial conceptual category in learning science. In the 1960s, Herbert Walberg and Rudolf Moss began to study learning environment, and developed the early version of the learning environment inventory and classroom environment scale. Fraser (1) and Peer and Fraser (2) conceptualize the learning environment in a specific way, and hold that the learning environment refers to all kinds of psychological, social and teaching situations that affect students’ academic performance, emotions and attitudes in the learning process. Magen-Nagar and Steinberger (3) from the perspective of constructivism, defined the learning environment as “the environmental atmosphere and atmosphere related to the behavior of teachers and students, which can take learning as a positive knowledge construction process,” emphasizing that the learning environment should support students’ autonomous learning and cooperative interaction with peers and teachers, so as to cultivate students’ initiative and innovation in the learning process (4).

The clinical learning environment integrates all kinds of factors that affect the learning effect of students in the learning process, including hospital culture, teaching staff, doctor-patient relationship, teaching resources, learning opportunities, and other clinical staff. It is an interactive network composed of all influencing factors, which are interrelated and interactive (5, 6). The clinical learning environment provides students with opportunities to apply and practice theoretical knowledge in a real environment, realizes students’ professional socialization, builds up students’ professional confidence, and promotes students’ career role transformation (7, 8). Compared with the general learning environment, the clinical learning environment is more closely integrated with the social environment, which has certain particularity and complexity, and is difficult for teachers to predict and control. At the same time, the clinical learning environment is a working scene with a clear service object, and students are not the core subject, which is also significantly different from the traditional school learning environment.

The clinical learning environment will have a significant impact on nursing students’ caring ability (9), communication ability (10), academic participation (11), humanistic care behavior (12), academic motivation (13), career readiness (14), and so on, which must be highly concerned by educators. Conducting a clinical learning environment assessment can ensure the quality of clinical internships for nursing students, enhance their sense of professional identity, and have a significant impact on improving the quality of nursing services and patient satisfaction. In order to evaluate the clinical learning environment of nursing students and promote the development of relevant empirical investigation, scholars have developed some measurement tools of the clinical learning environment of nursing students. This paper will review the research progress of the measurement tools of clinical learning environment for nursing students, and provide a reference for promoting the evaluation of clinical teaching environment for nursing students.

## Materials and methods

This review includes a systematic search, research review, and descriptive analysis of existing literature.

### Search strategy

In September 2021, three databases, including Web of Science (WOS), PubMed, and China National Knowledge Infrastructure (CNKI) (15), were searched with “nursing student” and “Clinical learning environment” as key search terms.

### Inclusion and exclusion criteria

#### Inclusion criteria

Relevance to clinical learning environment: The study must focus on measuring the clinical learning environment specifically for nursing students, using established tools or instruments designed for this purpose.
Tool evaluation: The study should describe or evaluate tools that assess various dimensions of the clinical learning environment, such as supervision, feedback, organizational support, or student satisfaction.
Publication date: Papers published before September 2021.
Type of study: Quantitative or mixed methods.
Publication language: Unlimited.

#### Exclusion criteria

Irrelevant subject matter: Studies not focused on nursing students or not concerned with clinical learning environments were excluded (e.g., studies that focus on educational theory without practical application in clinical settings).
Overlap with previous studies: Studies with identical or nearly identical research questions, samples, and tools as those already included in the review were excluded to prevent redundancy and ensure the inclusion of new insights.
Missing or incomplete data: Studies with incomplete or missing data, such as those without full descriptions of the tools used or inadequate reporting on measurement outcomes, were excluded to ensure the reliability and comprehensiveness of the review.

### Screening process

The authors screened the retrieved papers to assess their relevance. Only those related to the research purpose and that met the inclusion criteria were included. We screened the abstracts of articles with relevant titles, and if deemed applicable, the full text was retrieved and reviewed. A total of 19 articles were selected for further analysis.

### Charting the data

Microsoft Excel created a data chart describing the literature on the measurement tools. Data were extracted independently from the articles and classified according to the following headings: title, author, compilation time, initial sample, scoring method, dimension (entry type), and internal consistency.

### Collating, summarizing and reporting the results

We conducted an overall analysis of the research on clinical learning environment measurement tools for nursing students by combining all relevant findings from data retrieved from various sources to evaluate the existing research status and confirm the knowledge gap, which can provide a reference for future research.

## Result

### Clinical Learning Environment Scale

Dunn and Burnett (16), Queensland University of Technology, developed the Clinical Learning Environment Scale (CLES) in 1995. The compiling process of this scale is as follows: First, 12 nursing education experts revised the Ward Learning Climate Survey compiled by Orton and completed the first draft containing 55 items. The second test was conducted with 83 Australian nursing clinical instructors and 423 nursing undergraduates and clinical nurses as a sample group. Finally, through exploratory factor analysis and confirmatory factor analysis, we finally form “Staff-Student Relationships,” “Nurse Manager Commitment,” “Patient Relationships” and “Interpersonal Relationships” “and” Student Satisfaction “are a formal scale with 23 items in five dimensions, scored by Likert 5-point method. Cronbach’s alpha for five dimensions is 0.63–0.85. With the use of the CLE Scale, educators may evaluate emotionally relevant aspects of the clinical learning environment with accuracy and dependability, focusing resources on areas that require development.

### Student Evaluation of Clinical Education Environment Inventory

Sand-Jecklin (17), University of Virginia, developed the Student Evaluation of Clinical Education Environment Inventory (SECEE) in 2000. Interviews with a group of nursing teachers and a group of nursing students were conducted to determine “What are the important factors influencing student learning in a clinical setting?” Based on literature interviews and expert suggestions, the preliminary draft of the questionnaire was preliminarily established, and the students were measured many times with the compiled scale and open questions, and constantly supplemented and improved. Finally, the “Communication/Feedback,” “Learning Opportunities,” “Learning Support/Assistance” and “Department” are formed. The formal questionnaire composed of 29 items in four dimensions of Atmosphere was tested on 319 nursing students from four American nursing schools, with Cronbach’s alpha ranging from 0.89–0.94. Later, Sand-Jecklin (18) modified the scale twice and formed the modified version of the scale. The new scale consists of the “Instructor Facilitation of Learning Scale,” “Preceptor Facilitation of Learning Scale” and “Learning Opportunities Scale,” and contains a total of 32 items. Scores were scored by Likert 5-point method. Cronbach’s alpha was 0.82–0.94.

### Clinical Learning Environment Inventory

Chan (19), Chinese University of Hong Kong, developed the Clinical Learning Environment Inventory (CLEI) in 2001. In this scale, the author is concerned that the clinical learning environment is a multidimensional entity with a complex social background, based on the MOOS social environment theory, which not only pays attention to the actual environment but also the match between students’ preferences and the actual environment. In the compilation process, firstly, based on the College and University Classroom Environment Inventory, the scale draft is developed. Second, five university nursing experts and five clinical experts were invited to verify the questionnaire’s contents by checking the items’ adequacy and appropriateness. Finally, a pilot study with 20 nursing students, Formed a scale with 42 items in six dimensions, including “Individualization,” “Involvement,” “Task orientation,” “Innovation,” “Satisfaction” and “Personalization.” The Likert 4 scoring method was used. After the scale was developed, 108 Australian second-year nursing students were used as test subjects for reliability and validity tests. Cronbach’s alpha of six dimensions was 0.73–0.84, and the correlation with other scales was 0.39–0.47. CLEI was developed based on the existing scale of the College and University Classroom Environment Survey (CUCEI), taking into account the specific identity of the study subjects and ensuring that the scale is suitable for the higher education environment. In 2011, Salamonson et al. (20) from the University of Western Sydney adapted the CLEI scale, choosing only two factors around “clinical tutor’s support for learning” and “student’s satisfaction with clinical practice.” A simplified version of the scale CLEI-19, consisting of 19 items from three dimensions, “Satisfaction,” “Personalization” and “Clinical Facilitator,” was formed and scored by Likert 5-point method. Cronbach’s alpha for the three dimensions is 0.92–0.94.

### Clinical Learning Environment and Supervision Instrument

Saarikoski et al. (21) of the University of Applied Sciences, Turku, Finland, Clinical Learning Environment and Supervision Instrument (CLESI) in 2002. The scale includes “Ward Atmosphere,” “Premises of Nursing,” “Premises of Learning,” “Leadership Style of the Ward Manager” and “Supervisory” There are 27 items in five dimensions of Relationship, scored by the Likert 5-point method. The reliability and validity test is conducted through the empirical test of 416 Finnish nursing students. Cronbach’s alpha of five dimensions is 0.73–0.94. In 2008, Saarikoski et al. (22) adapted and completed the (Clinical Learning Environment Assessment Scale on the basis of CLESI. Supervision and Nurse Teacher Evaluation Scale, CLES+T), the new scale pays more attention to nursing teachers in clinical teaching Settings, Contains “Pedagogical Atmosphere on the Ward,” “Role of Nurse Teacher,” “Premises of Nursing on the Ward,” “Leadership Style of the Ward Manager,” “Supervisory Relationship” includes five dimensions and 34 items, scored by Likert 5-point method, and tested the reliability and validity of 549 Finnish nursing students. Cronbach’s alpha for five dimensions is 0.70–0.97. The CLES+T scale has attracted a lot of attention internationally, Wang et al. (23), Mueller et al. (24), Kim et al. (25), Al-Anazi et al. (26), Iyigun et al. (27), Ziba et al. (28), Johannessen et al. (29), and Sommers et al. (30) have translated the scale into their own languages and conducted cross-cultural debugging, and introduced the scale into Austria, South Korea, Saudi Arabia, Turkey, Ghana and other countries.

### Clinical Learning Environment Scale for Nursing

Zhu (31), Chinese Medical Sciences University, developed the Clinical Learning Environment Scale for Nursing (CLESN) in 2005. Initially, the dimensions and items of the scale were constructed by referring to existing kinds of literature, and the draft of the scale was formed through screening and evaluation by a group of students and experts. Forty-two items, including six dimensions of “interpersonal relationship,” “working atmosphere and team culture,” “student participation,” “task orientation,” “innovation” and “personalization,” were scored using Likert 5 points. After the scale was compiled, 248 nursing undergraduates from seven nursing colleges in China were used as test subjects to test the reliability and validity of the scale. The Cronbach’s alpha of six dimensions was 0.871 to 0.927, the retest reliability was 0.769 to 0.868, and the KMO was 0.934.

### Undergraduate Clinical Education Environment Measure

Strand et al. (32), Lund University in Sweden, developed the Undergraduate Clinical Education Environment Measure (UCEEM) in 2013. The scale focused on contemporary workplace learning theories and, based on literature review, semi-structured focus group interviews, and individual interviews, initially formed a list of five core themes and 45 items. Feedback from interviews with 15 stakeholders was obtained, 38 preliminary entries were formed, 77 medical students underwent pre-testing, and 463 medical students underwent measurement and evaluation. A scale consisting of four dimensions, “Quality of Supervision,” “Preparedness for Student Entry,” “Workplace Interaction Patterns and Student Inclusion,” and “Equal treatment,” with 25 items, was ultimately formed. The Cronbach’s alpha for these four dimensions was 0.79–0.91. Although the scale was developed with medical students as the initial sample, scholars such as Sharifipour et al. (33) and Chun et al. (34) have also applied the scale to surveys of nursing students in Iran and South Korea, verifying its applicability among nursing students.

The detailed information of measurement tools is shown in Table 1.

**Table 1:** Summary analysis of measuring tools.

Name of the tools	Initial sample	Author	Compilation time	Scoring method	Dimensions (number of entries)	Internal consistency
Clinical Learning Environment Scale (CLES)	Australian nursing students	Dunn	1995	Likert 5	Staff-Student Relationships (4), Nurse Manager Commitment (5), Patient Relationships (4), Interpersonal Relationships (6), Student Satisfaction (4)	0.63–0.85
Student Evaluation of Clinical Education Environment inventory (SECEE)	American nursing students	Sand-Jecklin	2000	Likert 5	Communication/Feedback (7), Learning Opportunities (8), Learning Support/Assistance (8), Department (6)	0.89–0.94
Clinical Learning Environment Inventory (CLEI)	Australian nursing students	Chan	2001	Likert 4	Individualization (7), Involvement (7), Task orientation (7), Innovation (7), Satisfaction (7), Personalization (7)	0.73–0.84
Clinical Learning Environment and Supervision instrument (CLESI)	Finnish nursing students	Saarikoski	2002	Likert 5	Ward Atmosphere (5), Premises of Nursing (4), Premises of Learning (6), Leadership Style of the Ward Manager (4), Supervisory (8)	0.73–0.94
Clinical Learning Environment Scale for Nursing (CLESN)	Chinese nursing students	Zhu	2005	Likert 5	Interpersonal Relationship (7), Working Atmosphere and Team Culture (7), Student Participation (7), Task Orientation (7), Innovation (7), Personalization (7)	0.87–0.93
Clinical Learning Environment, Supervision and Nurse Teacher Scale (CLES+T)	Finnish nursing students	Saarikoski	2008	Likert 5	Pedagogical Atmosphere on the Ward (9), Role of Nurse Teacher (9), Premises of Nursing on the Ward (4), Leadership Style of the Ward Manager (4), Supervisory Relationship (8)	0.70–0.97
Student Evaluation of Clinical Education Environment inventory (SECEE V3)	American nursing students	Sand-Jecklin	2009	Likert 5	Instructor Facilitation of Learning (11), Preceptor Facilitation of Learning (11), Learning Opportunities (10)	0.82–0.94
Clinical Learning Environment Inventory (CLEI-19)	Australian nursing students	Salamonson	2011	Likert 4	Satisfaction (7), Personalization (7), Clinical Facilitator (5)	0.92–0.94
Undergraduate Clinical Education Environment Measure (UCEEM)	Swedish medical students	Strand	2013	Likert 5	Quality of Supervision (11), Preparedness for Student Entry (6), Workplace Interaction Patterns and Student Inclusion (6), Equal Treatment (2)	0.79–0.91

## Discussion

The clinical learning environment is a multi-dimensional concept with rich connotations, which includes all the factors affecting students’ clinical learning and is of great significance to students’ clinical practice. Nursing is essentially a practical discipline, and clinical practice is the core link of nursing students’ training. Therefore, great attention must be paid to the clinical learning environment. In order to measure the clinical learning environment of nursing students, scholars have developed several tools. In the process of developing these measurement tools, researchers have carried out a large number of interviews, observed the actual situation of clinical learning, and also made good use of the Fraser classroom assessment method, MOOS human-environment interaction framework, workplace learning theory, and so on. All these have greatly improved the scientificity of the scale, especially some of the scales that have been concerned by many countries (regions), translated into many languages, and widely used, and these completed scales provide an essential reference for the evaluation of the clinical learning environment of nursing students.

However, these tools also have some limitations. The Clinical Learning Environment Scale (CLES), while widely used to assess the clinical learning environment, has several limitations. It tends to overlook the complexity and variability of real-world healthcare settings, such as differences in hospital size, resource availability, and patient demographics. For instance, larger teaching hospitals may offer more structured learning opportunities, while smaller or rural settings may provide fewer resources, which the CLES does not fully account for. It also fails to measure how these environmental factors—along with emotional and psychological support for students—affect learning outcomes, particularly in diverse or high-stress settings. The Student Evaluation of Clinical Education Environment Inventory (SECEE) is a useful tool for assessing the clinical education environment, but it focuses primarily on student perceptions of the learning environment, potentially overlooking more objective measures of educational quality, such as specific skill development or clinical outcomes. The tool has limited consideration of emotional and psychological support, which is critical in high-stress clinical settings where students face complex, emotionally demanding situations. The Clinical Learning Environment Inventory (CLEI) is primarily focuses on general factors like organizational support, educational quality, and feedback, without fully accounting for the variability across different clinical settings. It tends to overlook the emotional and psychological aspects of the learning experience, such as stress management or peer support, which are vital in challenging clinical environments. Finally, the tool may not fully reflect the complexity of interprofessional collaboration. The Clinical Learning Environment and Supervision Instrument (CLESI) tends to focus more on the quality of supervision and feedback, without fully addressing the broader complexities of clinical practice, such as interprofessional collaboration or the emotional and psychological support students may need. The instrument may not adequately capture the variability in learning experiences across different clinical specialties or settings, limiting its generalizability. The Clinical Learning Environment Scale for Nursing (CLESN) is a useful tool for evaluating the clinical learning environment in nursing education. The CLESN also tends to focus on students’ perceptions of supervision and support, without adequately addressing other critical factors like technological integration in clinical practice, interprofessional collaboration, or emotional and psychological challenges faced by students. These gaps limit the tool’s ability to capture the full complexity of modern clinical learning environments. The Undergraduate Clinical Education Environment Measure (UCEEM) is valuable for assessing clinical education environments, but it has notable limitations. Primarily, it focuses on general aspects of the learning experience, such as student engagement and supervision, without fully considering the variability in clinical settings. Additionally, the UCEEM does not adequately capture emotional and psychological support for students, which is critical in high-pressure environments. Finally, while it measures aspects of the educational climate, it does not thoroughly assess the impact of interprofessional collaboration or the dynamic nature of clinical practice, potentially limiting its ability to reflect the full scope of modern healthcare education.

In addition, we should see that although scholars have recognized the richness of the content of the clinical learning environment, including the “physical space of the hospital,” “psychological and interactive factors,” “organizational culture of the hospital,” “learning atmosphere of the hospital,” “clinical teachers,” “student satisfaction” and many other aspects, but at the specific measurement level. It is necessary to make the focus of attention clear according to the core content of the study. Frequent public health events significantly impact the entire medical environment, putting higher requirements for hospital emergency management, nosocomial infection prevention and control, risk assessment, student protection, teaching organization, etc. Kells and Mathis (35) pointed out that the impact of COVID-19 on nursing students should not be ignored, and more mental health services should be given to students in clinical teaching. In 2020, the publication of “IWA 35:2020: Quality of Learning Environments for Students in Healthcare Professions—Requirements for The Healthcare Education Providers in Care Settings” (36) by the International Organization for Standardization provides new guidance for our understanding of the clinical learning environment.

The ongoing effects of the COVID-19 pandemic have profoundly shifted the landscape of clinical learning. During the pandemic, clinical education faced unprecedented disruptions, including the suspension of in-person clinical rotations, increased reliance on virtual learning, and a surge in the use of simulation-based training. Many of the tools previously employed to assess clinical learning environments were not designed to capture the nuances of these new, virtual or hybrid environments. Furthermore, the abrupt shift to online learning raised questions about how well students were engaging with the material and whether they were receiving adequate feedback and mentorship from instructors. A critical examination of existing tools reveals that many of them struggle to evaluate this new context effectively. Tools that measure aspects like “clinical exposure” or “student-instructor interaction” might not fully encompass the challenges of online learning or the altered dynamics in virtual learning environments. It’s essential for tools to incorporate measures that reflect these shifts, assessing not only knowledge acquisition but also the emotional and social dimensions of remote learning, which were previously overlooked. The development of artificial intelligence has also put forward new requirements for traditional clinical teaching. Promoting the integration of virtual and real practical teaching, allowing students to directly use artificial intelligence technology for medical project practice and exploration, and applying artificial intelligence to clinical teaching scenarios is an important trend for the future development of medical education. Learning based on digital learning platforms and artificial intelligence will become an important component of evaluating clinical learning environments and requires more attention.

Therefore, on the basis of carefully combing and analyzing the existing research on clinical learning environment measurement tools for nursing students, paying attention to the development of the actual health industry, and adapting the existing clinical learning environment measurement tools can better promote the development of relevant empirical investigations.

## Conclusion

This review contains a wealth of research articles on measuring the clinical learning environment of nursing students. These articles trace the development process of measuring tools for the clinical learning environment of nursing students, which is of great value for better conducting clinical learning environment assessment of nursing students and can provide reference for scholars to conduct related research and practice. At the same time, this review also proposes some improvement areas in this research field and looks forward to the research prospects in this field in order to inspire future research.

## Data Availability

The original contributions presented in the study are included in the article/supplementary material, further inquiries can be directed to the corresponding authors.
